# On the Possibility of Predicting Glycaemia ‘On the Fly’ with Constrained IoT Devices in Type 1 Diabetes Mellitus Patients

**DOI:** 10.3390/s19204538

**Published:** 2019-10-18

**Authors:** Ignacio Rodríguez-Rodríguez, José-Víctor Rodríguez, Ioannis Chatzigiannakis, Miguel Ángel Zamora Izquierdo

**Affiliations:** 1 Departamento de Ingeniería de la Información y las Comunicaciones, Universidad de Murcia, Facultad de Informática, 30100 Murcia, Spain; mzamora@um.es; 2 Departamento de Tecnologías de la Información y las Comunicaciones, Universidad Politécnica de Cartagena, 30202 Cartagena, Spain; jvictor.rodriguez@upct.es; 3 Dipartimento di Ingegneria Informatica Automatica e Gestionale ‘Antonio Ruberti’, Sapienza Università di Roma, 00185 Roma, Italy; ichatz@diag.uniroma1.it

**Keywords:** continuous glucose monitoring, wearable devices, constrained devices, time series forecasting, machine learning

## Abstract

Type 1 Diabetes Mellitus (DM1) patients are used to checking their blood glucose levels several times per day through finger sticks and, by subjectively handling this information, to try to predict their future glycaemia in order to choose a proper strategy to keep their glucose levels under control, in terms of insulin dosages and other factors. However, recent Internet of Things (IoT) devices and novel biosensors have allowed the continuous collection of the value of the glucose level by means of Continuous Glucose Monitoring (CGM) so that, with the proper Machine Learning (ML) algorithms, glucose evolution can be modeled, thus permitting a forecast of this variable. On the other hand, glycaemia dynamics require that such a model be user-centric and should be recalculated continuously in order to reflect the exact status of the patient, i.e., an ‘on-the-fly’ approach. In order to avoid, for example, the risk of being disconnected from the Internet, it would be ideal if this task could be performed locally in constrained devices like smartphones, but this would only be feasible if the execution times were fast enough. Therefore, in order to analyze if such a possibility is viable or not, an extensive, passive, CGM study has been carried out with 25 DM1 patients in order to build a solid dataset. Then, some well-known univariate algorithms have been executed in a desktop computer (as a reference) and two constrained devices: a smartphone and a Raspberry Pi, taking into account only past glycaemia data to forecast glucose levels. The results indicate that it is possible to forecast, in a smartphone, a 15-min horizon with a Root Mean Squared Error (RMSE) of 11.65 mg/dL in just 16.15 s, employing a 10-min sampling of the past 6 h of data and the Random Forest algorithm. With the Raspberry Pi, the computational effort increases to 56.49 s assuming the previously mentioned parameters, but this can be improved to 34.89 s if Support Vector Machines are applied, achieving in this case an RMSE of 19.90 mg/dL. Thus, this paper concludes that local on-the-fly forecasting of glycaemia would be affordable with constrained devices.

## 1. Introduction

Diabetes is a disease characterized by high blood sugar levels as a consequence of the body’s inability to produce and/or use insulin. In a healthy human, glucose homeostasis represents a closed-loop system which is able to regulate blood glucose levels. In this way, the pancreas presents β cells which are sensitive to high glucose levels and produce insulin, a strong hormone able to reduce hyperglycemia in blood by allowing a “passage” for glucose to enter the cells.

This regulation is not naturally possible in Type 1 Diabetes Mellitus (DM1). Patients with a certain evolution of DM1 do not produce any insulin and must inject this hormone or wear an insulin pump in order to reduce their glucose levels. Furthermore, diabetic people need to check their glucose level several times per day and, based on these data as well as other factors like meals, exercise, and many others, try to predict the evolution of their glycaemia. Then, they have to decide how much insulin is required to keep their blood glucose level within a normal range (avoiding both hyper- and hypoglycemia). So, the possibility of accurately forecasting future blood glucose levels is an important task in order to infer insulin dosages.

Fortunately, new technological possibilities offer a completely new horizon in diabetes management. Nowadays, it is not possible to think about the future of the control of diabetes without the concept of Continuous Glucose Monitoring (CGM). This concept has led to a revolution in diabetes care, since it continuously provides the magnitude, tendency, frequency and duration of the fluctuations of glucose levels. Compared to conventional glucose monitoring (finger sticks), which allows only between three to ten measurements per day, CGM-based devices deliver up to one measurement per minute (1440 day). Such data could potentially help in better predicting the magnitude, tendency, frequency and duration of the fluctuations of the glucose level [1,2].

Due to the importance of diabetes, the design of systems for its management has been widely studied. Although so-called portable glucose prediction models (which are calibrated offline) have been proposed, as shown in [3,4], when it comes to assuring a robust and continuous glucose prediction process in DM1 patients (which is essential) in the event of loss of connectivity, wearable solutions that both model and forecast locally have to be considered. This way, a critical aspect of such existing wearable solutions for diabetes management [5] is their lack of computational power to locally process the CGM data and predict future blood glucose levels [6]. The collected data are transmitted to a nearby gateway device that relays the recording to the cloud, where advanced analysis algorithms are executed [7] and [8]. Due to the fact that glycaemia can change pretty quickly in DM1 patients, and also because of unexpected changes in their daily routine, it is important to monitor the data continuously in order to predict future blood glucose levels. Depending on the sampling frequency of the CGM sensor, the continuous transmission of data over a wireless network affects the battery performance of the CGM sensor, as do the characteristics of the nearby gateway.

The underlying assumption behind the research reported here is that if wearable devices become capable of executing forecasting algorithms that can process in real time the data collected from the CGM sensor and generate alerts for the DM1 patient without relying on the cloud infrastructure, then they would conserve battery power and minimize memory requirements. The concept of combining the resource-bound last-mile sensors of any IoT-related application with computational capabilities is receiving increasing attention from researchers and practitioners.

In contrast to remote prediction, such a local analysis and forecasting has the advantage of being independent of an internet connection, which could be unavailable under some circumstances (remote places, general failure of the phone coverage, etc.), or if the device needs to be in flight mode and cannot gather data from the cloud (cinema, conference, or airplane). This idea of forecasting glycaemia locally in DM1 has been recently explored in [9], where a previously obtained model is executed in a smartphone (in a time of 7 ms), therefore proving the possibility of using constrained devices for this task.

Moreover, processing the data locally also increases the level of control over the data. Since the data collected from the wearable devices is not forwarded to the cloud, we are in a position to allow the user to maintain control of all the collected data. This idea reinforces the goal of this research, of guaranteeing the privacy of confidential data. Data privacy is a crucial factor in designing smart healthcare systems. On the other hand, remote computing allows for the introduction of more complex models that could better express glucose dynamics, or permits the computation with more data in order to achieve more accurate results.

The idea of running highly demanding algorithms within a wearable device involves a challenge due to the restricted computational resources and available battery power. On the other hand, sending data to the cloud, although continuously using the wireless network, allows faster computation by a remote server. Therefore, these two options (local/remote forecasting) involve different computational environments. Some of the current options where glycaemia prediction can be performed, along with their corresponding processing powers, are detailed in Table 1.

Thus, depending on the device where we are computing the collected values, the limitations of the microprocessor could be restrictive and impose a specific algorithm and characteristics/number of data. Previous works have applied different methods to forecast glycaemia, some of them being more appropriate to a specific computational environment than others [10,11] and, in addition, it should be noted that the data processing requirements will be heavier as more variables are taken into account.

Because of this, in this paper, the computing tasks performed in constrained devices will consider only past blood glucose levels, thus avoiding the possible overcharging of the limited devices. Moreover, the fact of using just one monitoring device like the Freestyle Libre (and therefore obtaining just one variable) can be seen as an advantage for the user, since the patient only needs to wear and take care of one biosensor.

The aim of this paper is to perform glucose modeling and prediction in diabetic people by using only past glycaemia as the input feature (in order to alleviate the computational effort), and then make a fair comparison and discuss the pros and cons of executing glycaemia predictive algorithms (modeling and forecasting, i.e., both stages) locally in constrained devices or in a desktop computer. Moreover, a discussion about the computational burden obtained when varying some parameters like the sample frequency of blood glucose levels and the number of past used data, as well as how much accuracy can be achieved with different predictive horizons is presented. With this, we look for the possibility of doing this task locally, i.e., in a smartphone, which could be useful under some conditions (unstable internet connection, remote areas). The desktop computer will provide an order of magnitude.

As a result, although modeling and forecasting glycaemia using cloud computing present many advantages, we will evaluate if it could be possible to translate this double task to a constrained device (simplifying the work by using univariate approaches) in order to use the limited computational power existing in small devices under unavailability of the cloud resources.

This way, the different requirements of each case are also analyzed in terms of accuracy and computational effort —looking for a balance— so that an overview of the most suitable algorithm specifications is offered. Such an overview is proposed in terms of the appropriate sliding windows to perform the prediction (PSW), the achievable prediction horizons (PH), data sampling frequencies (SF) and, as results, Root Mean Square Error (RMSE) and Computer Effort (CE) (measured in seconds of computation). This last finding will determine if the forecasting task can be performed in a small device. For this, we have tested two portable devices (a Samsung smartphone and a Raspberry Pi), and will compare the results with those obtained using a desktop computer. The results indicate that it is possible to model and forecast, in a smartphone, a 15-min horizon with a RMSE of 11.65 mg/dL in just 16.15 s, employing a 10-min sampling of the past 6 h of data and the Random Forest algorithm. With the Raspberry Pi, the computational effort increases to 56.49 s assuming the previously mentioned parameters, but this can be improved to 34.89 s if Support Vector Machines are applied, achieving in this case an RMSE of 19.90 mg/dL. Thus, this paper concludes that local on-the-fly forecasting of glycaemia would be affordable with constrained devices.

This paper is structured as follows: after this introductory section, Section 2 reviews the literature on the prediction of glucose levels and their required computational effort. Section 3 describes the experiments and data collection. Section 4 characterizes the algorithms studied and the parameters used in this study. Section 5 presents the implementation of the software and hardware, which is followed by Section 6 with the results of the predictions and the computational burdens they imposed, depending on the device, leading to a discussion in Section 7. Finally, Section 8 concludes the paper offering some conclusions and possible future work.

## 2. Related Work: Algorithms to Predict Glycaemia in Diabetes Mellitus

### 2.1. Univariate Approaches

Although some deep learning models have recently demonstrated their capability to forecast glucose levels with an accuracy of RMSE = 9.38 ± 0.71 mg/dL over a 30-min horizon [12], in order to avoid the problems derived from overloading the execution of both the modeling and forecasting tasks in an ‘on-the-fly’ prediction (allowing, in this way, for such tasks to be achievable within a constrained device), only univariate options have been studied in the experimental stage of this work (those which take into account only past glycaemia). This is useful to reduce the complexity of the predicting algorithm and hence obtain a lower calculational burden. Other modeling approaches, like the use of differential equations, well studied by De Gaetano et al. [13], are remarkable but will be considered in a separated work carried out by the authors, being this paper focused only on machine learning techniques.

In the scientific literature, it is possible to find some approaches to predicting future glucose levels, with Artificial Neural Networks (ANNs), which use only glycaemia, as can be seen in Pérez Gandía et al. [14], who obtained a 45-min prediction including nothing more than glycaemia data in a neural network, with an acceptable error level. This method is to be discarded as it requires lots of data in order to calculate the vast number of weights. This takes a lot of training time and computational effort, and using only glycaemia, the risk of overfitting is clear.

Plis et al. [15] monitored five diabetic people for four days in 2014 and forecast glycaemia by using Autoregressive Integrated Moving Average (ARIMA) models, with PHs of 30 and 60 min, obtaining RMSEs of 22.9 and 42.2 mg/dL, respectively. These results were compared with those obtained by using SVM, offering RMSEs of 19.6 and 35.7 mg/dL. Although the latter performance is better, it should be noted that in the SVM, other physiological features were included. In 2007, Reifman et al. [3] studied 15 people with DM1 for 5 days, also using an autoregressive (AR) model and achieving accuracies of 85.3% at a PH of 30 min and 66.2% at 60 min. AR models, and especially ARIMA model, are the usual approaches chosen to forecast glycaemia by using only its past values.

Hamdi et al. [16] monitored 12 patients, and used a mixed algorithm with SVM and Differential Evolution (DE) algorithms, achieving a prediction with PHs of 15, 30, 45 and 60 min, obtaining RMSEs of 9.44, 10.78, 11.82 and 12.95, respectively, using past values of glycaemia. These results are better than the previously mentioned ones, but the inclusion of the DE part makes it difficult to replicate these results with different data.

In addition to these methods, it is also necessary to mention the Random Forest (RF) algorithm. Random decision forests are an ensemble learning method for classification, regression and also prediction tasks. It operates by constructing a multitude of decision trees at training time and outputting the class that is the mode of the classes (classification) or mean prediction (regression) of the individual trees. Random decision forests correct for decision trees’ habit of overfitting to their training set.

RF and SVM are used and compared in [17] (as well as decision trees and Naïve Bayes models) to predict hypoglycaemia using glycaemia and medication as features in an undetermined patient dataset, the first two (RF and SVM) being the best ones at predicting low levels of blood glucose, up to 97.5% and 97% of accuracy in a one-day window. The results of the other two models were significantly worse. Unfortunately, that paper is only focused on hypoglycaemia and cannot be generalized.

RF has been compared with ARIMA in the task of predicting time series, with good performance. Kane et al. [18] found that the RF method could improve the ARIMA results in this subject, in a study about avian influenza outbreaks, with a mean square error of 6.31 cases for RF compared to 26.95 cases for the ARIMA model.

So, to sum up, the ARIMA model has been widely used to predict glycaemia using only past values. There have been some approaches using SVM, although in some cases including also pulse variables like insulin dosages. In addition, RF has been used in the DM1 field, and it would be interesting to study its performance in glycaemia forecasting in order to see if it offers a good performance. Thus, these will be the three methods used in this paper to carry out the comparison: ARIMA, RF, and SVM.

Added to the fact of making a comparison, there is also the necessity to make that comparison in a standardized environment, as will be considered in this work. On the contrary, as was previously pointed out, some previous research was performed with the data of only five people, and others with twenty-eight patients. Some of them monitored for five days and others for only two. And, in some cases, the experimental stage was unclear. In the same vein, the discussion about the predictive possibilities is unequal. Not all the works studied the same PH, and in addition, although most of them presented their results in terms of RMSE, some others used the Continuous Glucose Error Grid Analysis (CG-EGA) and the Prediction Error Grid Analysis (PRED-EGA) [4]. In this sense, some of them are focused on analyzing accuracy when hypoglycaemia, since the limitations of RMSE in clinical prediction accuracy have been demonstrated, especially in the vicinity of low blood glucose values [19]. In any case, it is necessary to state that the use of RMSE in the present paper should be good enough for its main purpose, that is, the analysis of the computational burden that can be expected when modeling and predicting glycaemia in constrained devices. This way, an excessively ramified discussion is avoided. However, a deeper study regarding accuracy is the logical next step that is going to be addressed in future works of the authors.

It should be noted that the idea of analyzing the computational effort when executing a blood glucose level prediction model has already been addressed in a valuable previous work published by Naumova et al. [20] which is focused on the accuracy of the Fully Adaptive Regularized Learning (FARL) algorithm. In this case, the mentioned EGA criteria is considered. In the present work, we will concentrate our efforts on the study of the computational restrictions of constrained devices in the modeling and prediction of glucose levels in DM1 patients.

### 2.2. Computational Burden of the Algorithms

The glycaemia forecasting task needs to be performed in a specific hardware and, as it will be made several times per hour —‘on-the-fly’ (recalculating the model and then predicting)− it is necessary to take into account the execution time. This computing effort is an important constraint but, to the best of the authors’ knowledge, there is no previous work in diabetes management which studies or compares both computational time and accuracy of the prediction methods, obtaining a compromise solution.

Depending on the ML algorithm that we choose, we will face at some point a computer limitation, not only due to hardware, but also to software, including the Operating System [21]. This idea of restrictions in devices has been explored previously in some more demanding processes, like the Internet Protocol traffic flow [22]. In this case, some methods applied to the classification task were studied and compared.

It is possible to find an ML performance comparison in the field of medicine, as can be seen in [23], regarding Functional Magnetic Resonance Imaging (fMRI), which compares the accuracy of six different ML algorithms (among them, RF and SVM) applied to neuroimaging data of people engaged in a bivariate task, asserting their belief or disbelief of a variety of propositional statements. In this interesting study, RF was found to be more accurate than SVM. The performance of each algorithm was treated, reducing each feature set in order to reduce the amount of data to deal with, therefore lowering the computational burden without losing accuracy.

In [24], we can find a general comparison of ML algorithms, among them RF and SVM. In this case, the algorithms were tested using the free Gisette Dataset (https://archive.ics.uci.edu/ml/datasets/Gisette), a handwritten digit recognition problem. With this, SVM is one or multiple orders of magnitude faster, but RF is more precise.

Concluding with this review, to the best of the authors’ knowledge, no data about the computational burden have been found in glucose prediction for DM1; so, in this work, we firstly have the aim of improving the computational performance of different glycaemia forecasting methods by reducing the features considered to only one: the past values taken into account with a specific sample frequency (SF), this being a novelty of the present paper. This SF could even be modulated in a preprocessing step, prior to the ML algorithm execution. Then, the possibility of running such algorithms in constrained devices will be analyzed. With this, our results could be exportable to other similar ML applications to time series data, frequently used in medicine, for example.

## 3. Description of the Experiment

There are some remarkable experimental data sources, like the D1NAMO project [25], which implied monitoring 20 healthy subjects and nine patients by recording their ECGs, breathing, accelerometer signals as well as glucose levels. Moreover, there also exists the so-called T1D Exchange Biobank (https://t1dexchange.org/research/biobank/).

But in this paper the previous section presented the disparities of the conditions under which the previous work presented in the scientific literature have obtained their results. Hence, with the purpose of overcoming such limitations, a data-collecting experiment was carried out, in order to provide uniformity to the comparison which will be presented later. So, to obtain an empirical and complete set of features in the present work, a novel dataset has been achieved by means of an innovative monitoring campaign. For a period of up to 14 days, the glycaemia of 25 people was monitored continuously, while maintaining their normal life. The monitoring campaign was previously approved by the Ethical Research Commission of the University of Murcia on January 25th, 2018 (Id. 1683/2017).

The volunteers were all type 1 diabetic, with a basal-bolus treatment, using slow insulins such as Levemir, Tresiba, or Lantus—which achieve a flat-action curve— and fast insulin such as Humalog Lispro. The former involves more than 24 h as a basal coverage, and the latter is used to compensate for a rise of glycaemia, which can be due to an intake of a meal, or a hyperglycaemia caused by other reasons. All subjects gave their informed consent before they participated in the study.

The experimental group was composed by 14 men and 11 women, all of them under medical treatment and professional supervision. This monitoring stage was passive, without interfering with their treatment, all of them being encouraged to follow their doctor’s advice. The ages covered a range from 18 to 56 years old, the average being 24.51 years, most of them being young adults.

The patients were chosen with an illness evolution of at least five years, in order to be sure that they were familiar with the course of the disease.

All of them were fully informed about the purpose of the experiment, and were usually under well-controlled DM1, all of them presenting a glycated hemoglobin (HbA1c) between 6% and 7% at the beginning of the experiment.

All the patients declared that they led a healthy life, and all of them engaged in a sports activity at least three times per week. There has also been a control of the schedules, trying to ensure that all of them follow some kind of routine, without abrupt changes of daily timetable. In addition, they followed a balanced diet, according to their caloric necessities. The patients were encouraged to continue their usual habits and, in any case, follow the instructions of their endocrinologist.

The patients under study wore a CGM sensor: Freestyle Libre, from Abbott Company. This is a ground-breaking device consisting of a patch and a measurer, which allow patients to easily check their current glycaemia (not blood-glucose levels, but interstitial-glucose levels). The main characteristic of this gadget is that it not only permits checking glucose levels as often as desired, but it also registers data every minute. It should be said that it has constituted a little revolution in a way, because of its affordable price and its sufficient accuracy (11.4% Mean Absolute Relative Difference, MARD). In the measurer, the patients are invited to take note of fast-insulin dosages, slow-insulin dosages, as well as the equivalent carbohydrates of each meal, so that the data are no longer a personal, subjective evaluation.

This CGM has a maximum life of 14 days, but sometimes it falls prematurely, due to loss of adhesion, excess humidity, or just accidental separation, it not being possible to be reattached. This fact, added to the fact that the first days could show inaccuracies due to a lack of calibration, suggested taking into account just nine days out of the fourteen-day lifetime, discarding the first ones since they were the calibration period, and the last ones simply in case of not reaching the maximum expected lifetime. So, we considered 5400 h of data for our experimental phase.

Freestyle Libre is a Flash Glucose Monitoring device. This means that the glucose levels are collected and shown on demand, using Near Field Communication (NFC) and hence requiring an explicit gesture of the patient. However, some devices behave as transductors NFC-Bluetooth, (as the popular *miao-miao*; https://miaomiao.cool/?lang=en). This device is attached to the Libre sensor and allows sending periodically the data to the smartphone.

The monitoring campaign was took place in 2018 and was all the time under the supervision of the Endocrinology Department of the “Morales Meseguer” and “Virgen de la Arrixaca” Hospitals, both renowned institutions of the city of Murcia (Spain).

After obtaining all the data, the different values were pre-processed, cleaning outliers and gaps. Regarding the cleaning outliers issue, we used extreme value analysis, either using scatterplots or looking for values more than two times standard deviated from the mean. With respect to the gaps, we used interpolation methods, adding fingerstick glucose values if available. The data were stored complying with the strictest data protection rules protecting personal information. In addition, the Ethics Committee of the Universidad de Murcia supervised the patients’ monitoring.

The obtained dataset will be extremely useful in this and future research. In Table 2, different data regarding the population considered in the monitoring campaign can be seen.

## 4. Methods for Glucose Level Prediction

Our goal is to develop reliable prediction models that can estimate the future interstitial glucose level with high accuracy based only on current and past glycaemia data collected from the CGM sensor and evaluate the computational effort linked to this modeling and forecasting task in different hardware environments, with an emphasis on constrained devices. Our target considers merging the stages of building the model and forecasting due to the fact that glycaemia dynamics in DM1 sometimes changes pretty fast, and such circumstance implies the necessity of revising the model with the recent past PSW. This way, we have obtained patient-centric models by using different ML methods to capture the properties of the interstitial glucose time series of each individual patient. Therefore, for each patient, we have generated separate prediction models, i.e., one per each specific prediction.

**Figure 1:**
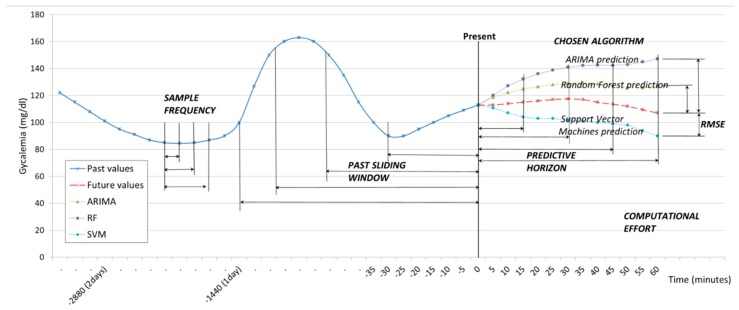
Schema of the parameters under discussion.

The data provided by the CGM sensor were sampled with SFs of one measurement every five minutes, ten minutes, and fifteen minutes. Therefore, the SF controls the velocity of the data that we are taking into account. The values sampled are used to create a past sliding window (PSW) which includes historic values, and which can vary from three to thirty-six hours. The PSW controls the volume of data the model uses for the prediction. Given the sliding window data, the model continuously predicts the glucose levels at preset PHs of 15, 30, 45 and 60 min ahead from the present time. All through the procedure, the time of execution is monitored, and this will be expressed as Computational Effort (CE), measured in seconds. These parameters are illustrated in Figure 1.

In more detail, at a given time t, the data collected from the CGM sensor over the previous period T are used to generate the training set of n data points ${\left\{x_{i}\right\}}_{i=1}^{n}$, where x_i_ ∊ R is each individual datapoint obtained from the CGM sensor. We say that n denotes the size of the data set and n/T the velocity of the data. Given this training set, the goal of the prediction model is to approximate the real underlying value by mapping accurately enough so that it can predict the next value of δ, that is, the output value y(t + δ) ∊ R for that time series. We say that δ is the PH.

The ‘on-the-fly’ approach is based on a sliding window, which works as follows. As soon as a new value of CGM is obtained, the training set is reorganized by removing the oldest observation (x_1_), shifting all the values one position up (i.e., x_i_ becomes x_i−1_) and finally adding the new value received as the newest one (x_n_). In this way, the dataset size and ordering of the observations is always preserved.

Although, as stated, computing in a more powerful device like a server could allow for the execution of more complex models, in order to carry out a fair comparison, the same univariate algorithms will be used. As previously mentioned, the methods considered in this work for glucose level prediction are the following:

(1) Autoregressive Integrated Moving Average (ARIMA): Univariate (single vector) ARIMA is a forecasting technique that projects the future values of a series based entirely on its own inertia [26]. ARIMA attempts to characterize the movements in a stationary time series as a function of a combination of autoregressive (AR), integration (I)–referring to the reverse process of differencing to produce the forecast–and moving average (MA) operations. The model is stated as ARIMA (p,d,q) representing the order of the autoregressive components (p), the number of differencing operators (d), and the highest order of the moving average term.
(2) Random Forest (RF): RF algorithms employ a technique known as bagging, whereby data instances are resampled multiple times to produce multiple training subsets from the training data [27]. Decision trees are then created from each training subset until ensembles of trees have been created. Each tree then casts a unit vote for the outcome of an incoming data instance class label. RF is flexible, with only limited computational resources required.
(3) Support Vector machines (SVM): The basic idea of SVM for time series approximation is mapping the data into a high-dimensional feature space by a nonlinear mapping and then performing a linear regression in the feature space [28]. The nonlinear mapping can be efficiently computed through a kernel function, without iterating over all the corresponding data points. Given the kernel function, the SVM learner tries to find a hyperplane that separates the positive data points from the negative ones and at the same time maximizes the separation (margin) between them. This method is known to be resilient to overfitting and to have good generalization performance, due to the max-margin criterion used during optimization. Furthermore, the SVM is guaranteed to converge to a global optimum due to the corresponding convex optimization formulation.

The prediction performance measure can be explained by the following: the performance of each different model is evaluated in relation to the size of the dataset (n), the data velocity (n/T) and the PH (δ). The values predicted by each user-centric models are compared with the actual values collected from the CGM sensor. The Root Mean Square Error (RMSE) is then used to evaluate the prediction accuracy, as this is the most broadly used metric in the relevant literature to measure prediction performance, as reported in Section 3. The RMSE is the square root of the mean of the squares of the differences between the predicted and the observed values.

## 5. Implementation

The initial models were implemented using the programming language R v3.5.0 in combination with the Classification and REgression Training (CARET) library v6.0-84. For the evaluation of the performance, firstly a desktop computer, equipped with an AMD Ryzen 7 1700X processor (octacore, two threads per core) was used, operating at 3.8 GHz, with 32 GB DDR4 RAM at 2666 MHz CL19. All 16 threads were used for the computations. These results will serve as a reference in order to estimate the results in a more powerful device. It should be pointed out that the RAM requirements during the experiment were at all times below the 100% of RAM usage, and also parallel computing was used.

After this, we compared these reference results with those obtained using two constrained devices: a smartphone and a Raspberry Pi.

The smartphone is a Samsung, model S8+ (https://www.samsung.com/global/galaxy/galaxy-s8/specs/), with an octacore Exynos 8895 system-on-chip (4 × 2.3 GHz M2 Mongoose & 4 × 1.7 GHz Cortex-A53 ‘GTS’) and 4 GB of LPDDR4 RAM 1794 MHz. This smartphone has been used previously in IoT approaches and numerical computing [29]. The computation was performed by using its internal storage of 64 GB UFS 2.1. Although the Samsung S8+ is an Android-based device (actually with Android 9.0 ‘Pie’), R software and its libraries were run using UserLAnd (https://github.com/CypherpunkArmory/UserLAnd/issues), an open-source app which allows running several Linux distributions. We used it as a Debian (https://www.debian.org/index.en.html) simulator. The smartphone carried out the calculations simultaneously with its real, daily use, so for that reason, the computation was reserved to seven out of the eight cores.

The second small device chosen was a Raspberry Pi 3b+ (https://www.raspberrypi.org/ products/raspberry-pi-3-model-b-plus/), a prominent cheap device developed to help computerization in emerging countries. Its affordable price and sufficiency of capabilities allow it to be a candidate for developing IoT approaches [30]. It uses a Broadcom BCM2837B0 SoC with a 1.4 GHz 64-bit quad-core ARM Cortex-A53 processor, and only 1 GB LPDDR2 RAM at 900 MHz. The computation was over a MicroSDHC 16GB UHS-1 Class 10, and R was run using Raspbian (https://www.raspbian.org/), a free operating system based on Debian optimized for the Raspberry Pi hardware. This time, all four cores were used for computational purposes.

## 6. Results

### 6.1. Results Regarding Dependence of RMSE and Computational Effort on the Past Sliding Window

**Figure 2:**
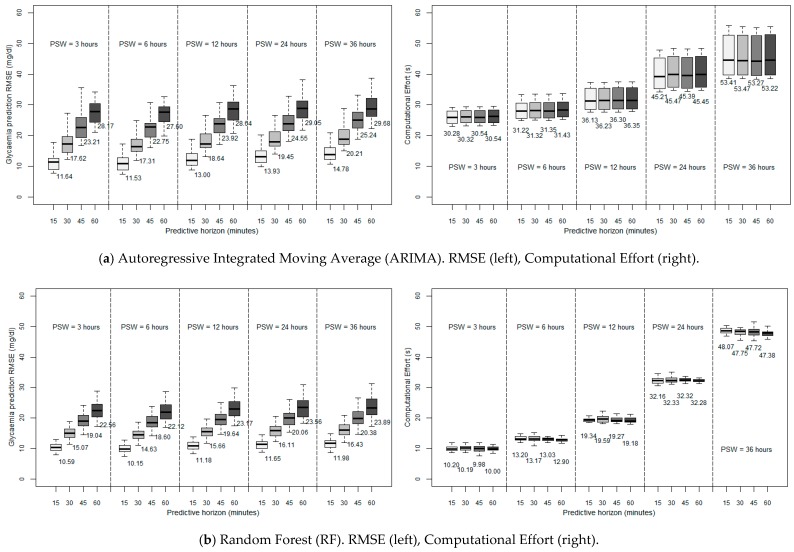

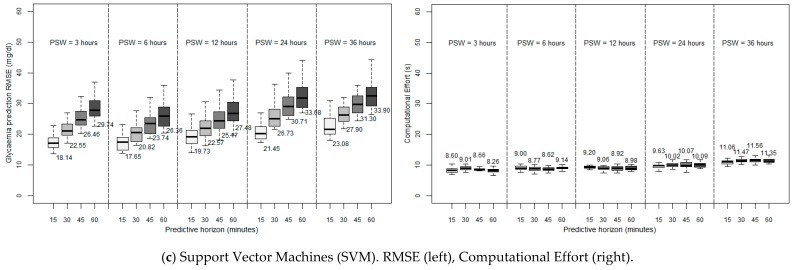
RMSE and Computational Effort of the different glycaemia prediction methods, for different PSWs and PHs, when executed in a desktop computer (all the simulations with an SF = 5 min). PH: Predictive Horizon (15, 30, 45, 60 min), PSW: Past Sliding Window (3, 6, 12, 24, 36 h).

**Figure 3:**
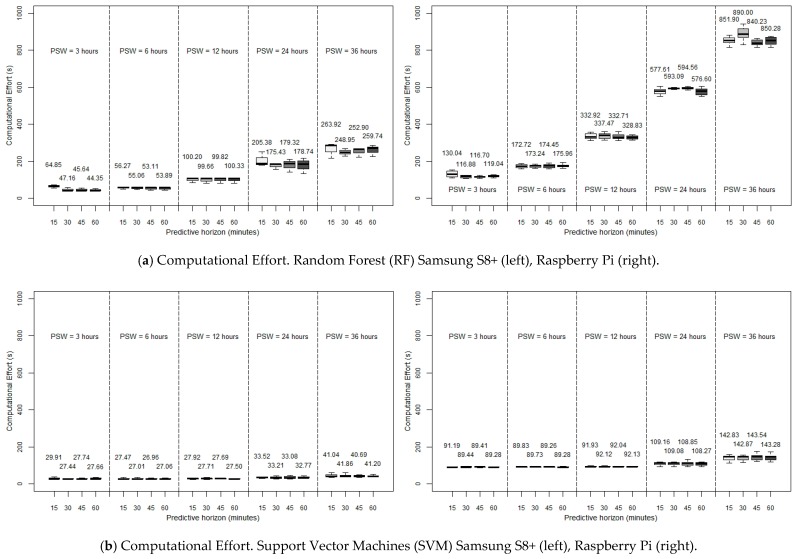
Computational Effort of the RF and SVM glycaemia prediction methods, for different PSWs and PHs, when executed in a Samsun S8+ smartphone (left) and a Raspberry Pi (right) (all the simulations with SF = 5 min). PH: Predictive Horizon (15, 30, 45, 60 min), PSW: Past Sliding Window (3, 6, 12, 24, 36 h).

The left side of Figure 2 shows the RMSE of the glycaemia prediction for the different ML techniques considered when executed in a desktop computer, as a function of the PSW and PH considered (with a SF of five minutes). Figure 3 depicts the computational performance of RF and SVM in the two constrained devices considered. Note the change of scale of the ordinate axis as compared to Figure 2 for the sake of clarity.

### 6.2. Results Regarding Dependence of RMSE and Computational Burden on Sample Frequency

**Figure 4:**
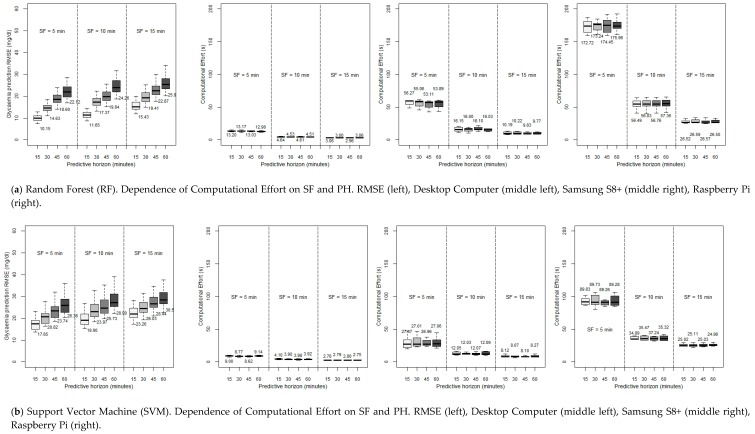
Computational Effort for the RF and SVM methods, for different SFs and PHs, when executed in a desktop computer and the two constrained devices considered (all the simulations with a PSW = 6 h). PH: Predictive Horizon (15, 30, 45, 60 min), SF: Sample Frequency (5, 10, 15 min).

The previous study was performed with attention to the PSW, with an SF of a registered measure each five minutes. Now, setting the PSW to be six hours (since this was the optimum value of the RMSE), we will try to lighten the computational burden by reducing the SF to ten minutes and fifteen minutes while at the same time maintaining an acceptable accuracy, that is, reducing the amount of data to deal with by one-half or even by two-thirds and, consequently, expecting a reduction in the CE at the cost of a foreseeable increase of the RMSE. In this sense, SF could be modulated prior monitoring by either adjusting CGM settings or using a transductor. Another possibility would be the use of data reduction methods (after collecting values) in order not to lose key data changes and fast fluctuations. This way, in Figure 4, the CE for the RF and SVM methods (again, ARIMA is discarded) as a function of SF is shown, in a desktop computer and the two constrained devices considered, for different PHs.

## 7. Discussion

### 7.1. Discussion Regarding Dependence of RMSE and Computational Effort on the Past Sliding Window

As can be observed, for all the algorithms, the error of the prediction is greater when larger PHs are employed, which seems reasonable, since the collected data is progressively further from the forecast. Generally, for the three algorithms, acceptable predictions are achieved for PHs of 15 min and 30 min (with average RMSEs practically smaller than 20 mg/dL for ARIMA and RF, and smaller than 28 mg/dL for SVM), the last two being accurate enough to provide an idea of the trend of the glycaemia. However, looking at the PSW, it can be seen that, for the three algorithms considered, the forecast is more accurate when using a PSW of six hours. So, this leads to the idea that there is a limit to moving backwards (six hours) when it comes to considering past data in order to improve the accuracy of the prediction, so that there is an optimum point beyond which past data stop being significant, and whose introduction causes more error in forecasting. Previous research has introduced this order of magnitude, linking it with the idea of circadian cycles [31] and the slots of morning/afternoon/night. All in all, focusing on the discussion of the algorithms, RF seems to be the most accurate one, achieving better predictions for all the PSWs and PHs considered. RF also presents a smaller standard deviation, which means that it is the method that is most independent of the specific characteristics of each patient: we must suppose that the particular routines and peculiarities of each subject affects the accuracy.

After these considerations regarding accuracy, if we want to implement these algorithms in a small device with limited computing power, we have to choose a light algorithm, in order to obtain in each iteration an affordable execution time, even sacrificing some accuracy. In Figure 2 (right side) we can see the performance of each algorithm developed in the above mentioned desktop computer. As can be seen, it is remarkable that the execution time is practically independent of the PH, the PSW being more influential and, as might be expected, for ARIMA and RF algorithms, the more we increase the PSW, the longer is the required time per each iteration since more data are considered and processed. However, regarding this issue, this time, SVM offers the best performance with another advantage: it is almost independent of the length of the PSW due to the capabilities of this hardware environment. On the other hand, both ARIMA and RF seem to increase exponentially the computational burden when expanding the PSW, making at some point the computation unaffordable. Anyway, the execution time seems to be affordable with the above-mentioned optimum PSW (six hours) for the three algorithms, and pretty similar to the smallest one (three hours), with values that range from 31.22 s (ARIMA) to 9.00 s (SVM) for a 15-min prediction. Therefore, we consider that in a desktop computer environment a good compromise is choosing RF as the algorithm with the best performance under these conditions since, as previously mentioned, it achieved the most accurate results with a small standard deviation.

As mentioned before, Figure 3 depicts the computational performance of RF and SVM in the two constrained devices considered. This time the ARIMA method is discarded because of its high requirements even in a desktop computer and for small PSWs. RMSE graphs are omitted because they present the same results as shown in Figure 2. In view of the results, it can be noticed that, in constrained devices, the data processing requirements soar, up the point that in RF, its exponential growth makes it unaffordable to use a Raspberry Pi for high PSWs: with a 36 h PSW, the 15-min ahead prediction takes more than 13 min to be calculated, which seems unreasonable for an ‘on-the-fly’ prediction. Focusing on the six-hour PSW, which, as previously mentioned, in the end is the most accurate window, we can observe a better performance with SVM, with a very fast execution with the smartphone (27.47 s for a 15-min prediction) as well as good enough with the Raspberry Pi (89.83 s). Continuing with this particular case (six-hour PSW), the execution time with RF is still acceptable in the Samsung S8+ (56.27 s for a PH of 15 min), but becomes inadequate with the Raspberry Pi (172.72 s for a PH of 15 min). In any case, it can be concluded that the possibility of executing glucose prediction methods in constrained devices can be considered as realistic.

### 7.2. Discussion Regarding Dependence of RMSE and Computational Burden on Sample Frequency

As can be observed, it is possible to see that our expectations were right. All the algorithms reduce their execution times when lowering the SF (especially when using a Rasperry Pi, where the improvement with this strategy is very remarkable). Specifically, when moving from SF = 5 min to SF = 15 min, RF decreases by more than 75% the execution times in a desktop computer (13.20 s to 3.06, in a 15-min forecast with RF, and from 9.00 to 2.76 s in the same case with SVM), and five times in constrained devices (56.27 s to 10.19 s for the Samsung S8+, in a PH of 15 min, and from the exaggerated 172.72 s to the restrained 26.52 s in a 15-min forecast in a Raspberry Pi). SVM, the fastest algorithm, also makes an improvement but it is not as prominent since it was already very fast before the SF variation; in any case, using a smartphone like the Samsung s8+, when moving from an SF of five minutes to an SF of fifteen minutes, the execution time decreases from 27.47 to 8.12 s (for a 15-min prediction), which is also an interesting point.

The preceding enhancement implies that the RMSE rises for each PH when the SF considered is lower (a higher value in minutes). This disadvantage is less remarkable when the PH is the biggest: in a PH of 15 min, when reducing the SF to one-third of its previous value, the RMSE goes up by around 50%: 10.15 to 15.43 mg/dL with RF, and from 17.65 to 23.26 mg/dL with SVM. But with the same change of SF, in a PH of 60 min, the RMSE increases by only around 10%–12% in both methods. So with this, the decision to reduce the SF depends on (in addition to our computationa3 power) how far ahead we want to predict the glycaemia.

In any case, the interesting point is that just by changing the SF from five minutes to ten minutes, still acceptable RMSEs are achieved while at the same time obtaining a considerable reduction in the CE, this reduction being more marked the less powerful is the hardware used. On the other hand, lowering the SF to one-third of its value results in the SVM, which offered a reasonable accuracy, reaches an unaffordable RMSE when the SF is equal to 15 min (23.26 mg/dL), although the CE is really low using a desktop computer, and pretty good with constrained devices. So, SVM is advised to be used with an SF of five minutes. But, even in this case, RF offers a better performance in every SF under discussion, with execution times in the same range when considering SFs of ten and even fifteen minutes.

## 8. Conclusions

In DM1 patients, glucose dynamics are influenced by the insulin dosage, diet, lifestyle, etc., and it is unstable and nonlinear [32]. It is therefore critical to achieve a sufficiently accurate forecast, and what is also important is to use a continuously updated model. This task, in an IoT environment, has been usually placed far in the cloud, but the risks of this approach are clear: a loss of connectivity blocks any possibility of continuing with the modeling and the prediction algorithm, so we need to ensure an ‘on-the-fly’ prediction as an overriding objective. So, the possibility of resolving this issue in a portable small device is a promising subject of study.

Therefore, we have conducted a comprehensive passive monitoring study involving 25 DM1 patients for 14 days. The obtained dataset is a novelty itself, since, to the best of the authors’ knowledge, this is the first time that CGM data have been registered for such a long duration in real-world conditions. The data allowed us to conduct a detailed comparative evaluation of glycaemia prediction models developed by using different ML methods: ARIMA, RF and SVM.

After a brief study about the accuracy, in which the past six hours were determined to be the most influential, and thus leading to the lowest RMSE, we replicated the calculations in three different hardware environments: first, a desktop computer, which served us as a reference, and two constrained devices: a smartphone (Samsung S8+) and a Raspberry Pi 3b+ (firstly, with an SF of five minutes). First of all, our evaluation indicates that ARIMA execution times are the longest in the desktop device (31.22 s for a 15-min PH, with a six-hour PSW), in comparison with RF and SVM (13.20 and 9.00 s, respectively). Then, focusing on the latter two algorithms, we have concluded that, in a desktop computer environment, it would be a good compromise to choose RF since it is the algorithm with the best performance while at the same time showing a small standard deviation. However, it is possible to see that RF has a marked exponential stress growth when considering big PSWs, which is more pronounced at lower powers of the computational environment. On the contrary, SVM is more independent of the value of the PSW. Regarding the execution of the algorithms in constrained devices, the data processing requirements soar but, in any case, in view of the affordable CEs obtained in some cases, especially with SVM (although still very high in the Raspberry Pi for RF and long PSWs), it can be concluded that the possibility of executing glucose prediction methods in constrained devices can be considered as realistic.

Focusing on the most accurate PSW, six hours, another analysis has been carried out: the possibility of lightening the forecasting task by reducing the SF. The results indicate that the previous sometimes unaffordable prediction times in constrained devices (when considering an SF of five minutes) gets reduced significantly when the SF is changed to ten or fifteen minutes. This makes it possible to forecast using a Raspberry Pi with RF, reducing the required computing time from 172.72 s in a 15-min PH, to 56.49 s just by sampling each ten minutes instead of each five minutes. The reduction in the smartphone Samsung S8+ in the same case is also impressive: from 56.27 s to 16.15 with RF (PH = 15 min), and from 27.47 to 12.05 s using SVM. On the other hand, RF suffers less from any lack of accuracy with this reduction of SF: from a 15-min PH, 10.15 mg/dL (SF = 5 min) to 11.65 mg/dL (SF = 10 min). The same change in SVM leads from 17.65 to 19.90 mg/dL.

Therefore, bearing all this in mind, it can be concluded that it seems that RF could be the best option for a small device, searching for a compromise in terms of speed and accuracy. A PSW of six hours, with an SF of ten minutes, allows reaching a really low RMSE in a fifteen-minute PH (16.15 s in the Samsung S8+, and 56.49 s employing the Raspberry Pi). This good performance is even maintained with a 60-min horizon. On the other hand, the calculational burden could be improved in the Rapsberry Pi by using SVM (SF = 10 min, PH = 15 min, CE = 34.80 s), but sacrificing accuracy.

Notwithstanding the foregoing, hybrid solutions can also be studied. There could be a hybrid interaction where some steps are carried out through cloud computing and others locally on the constrained device, i.e., transferring the modeling stage to the cloud when the battery of the constrained device is low, or, on the contrary, limiting the entire computational task in the small device when the internet connection is unstable. What is more, parameters like SF and PSW could be changed according to the situation: sampling with a greater SF value under glycaemic stability periods (therefore lightening the computation time of the modeling and forecasting tasks), and decreasing the SF value when necessary (the glycaemia is changing fast and a more accurate prediction is required). This way, an adaptable strategy would strengthen the reliability and good performance of the system in every different situation.

In this way, we have provided concrete evidence that constrained devices can be used for forecasting glycaemia locally and, therefore, wearable devices: (a) do not need to use large memory units to increase the volume of the stored data, and (b) they do not need to use high sample frequencies for collecting data from the sensors. This way, the hardware requirements can be optimized as well as the power consumption of the next generation of wearable devices, thus delivering products with (a) smaller size, and therefore more lightweight, and (b) a longer lifetime, thus requiring less maintenance from the end-users.

In future works, the authors will explore the possibilities of replicating the present study using Deep Learning, that is, introducing compressed previously-trained models in the constrained devices, since LSTM architectures can ease the execution in this type of scenarios. In addition to this approach, the authors also want to investigate the use of mathematical models —by using differential equations— in the prediction of glucose curves, which, as stated before, has already been analyzed [13].

Future research will be focused on the idea of introducing the performance and execution of complex models implemented on the cloud in the comparison with constrained devices, as well as the possible improvement of the obtained good performances by considering more features in this ‘on-the-fly’ modeling and prediction, such as, e.g., exercise, heart rate, and, of course, insulin and meals, in order to check if the accuracy can be improved without an unaffordable increase of the computational requirements.

## Figures and Tables

**Table 1:** Computational environments where glycaemia predictive algorithms can be implemented.

Device	Micro	Model	Speed	Cores	Number Cores/Threads	Architecture	Memory Type
Wearable.	Snapdragon Wear 3100 ^1^	Huawei Watch 2.	1.2 GHz.	4 × ARM Cortex A7.	4.	32-bit.	LPDDR3-400.
Raspberry.	BCM49408 ^2^	Pi 4.	1.8 GHz.	4 × ARM B53.	4.	64-bit.	DDR3-1600.
Smartphone.	Samsung Exynos 9 Serie 9820 ^3^	Samsung Galaxy S10.	2.7 GHz.	Dual-core Custom CPU + Dual-core (Cortex-A75) + Quad-core (Cortex-A55).	8.	64-bit.	LPDDR4-1794.
Desk Computer.	Intel Core i9 ^4^		3.6 GHz.	1 × i9-9900KF.	8/16.	64-bit.	DDR4-2666.
Server.	Intel Xeon ^5^		3.8 GHz.	1 × Intel Xeon W-3175X Processor.	28/56.	64-bit.	DDR4-2666.

^1^https://www.qualcomm.com/products/snapdragon-wear-3100-platform; ^2^https://www.broadcom.com/products/wireless/wireless-lan-infrastructure/bcm49408; ^3^https://www.samsung.com/semiconductor/minisite/exynos/products/mobileprocessor/exynos-9-series-9820/; ^4^https://ark.intel.com/es/products/190887/Intel-Core-i9-9900KF-Processor-16M-Cache-up-to-5-00-GHz-; ^5^https://ark.intel.com/es/products/189452/Intel-Xeon-W-3175X-Processor-38-5M-Cache-3-10-GHz.

**Table 2:** Data regarding the patients considered in the study.

Population Feature	Value		
Subjects (Number)	25		
Sex	14 men–11 women		
Occupation	16 students–9 office workers		
Population Feature	Median	Min	Max
Age (years)	24.51	18	56
Body Mass Index (BMI, kg/m^2^)	22.20	19.42	24.80
Duration of diabetes (years).	9	5	29
Fingersticks per day.	7	5	12
Insulin units per day (fast insulin + slow insulin, median).	47	36	59
HbA1C (%).	6.8	6.3	7.8

## References

[1] Phillip M., Battelino T., Atlas E., Kordonouri O., Bratina N., Miller S., Biester T., Stefanija M.A., Muller I., Nimri R. (2013). Nocturnal glucose control with an artificial pancreas at a diabetes camp. N. Engl. J. Med..

[2] Reifman J., Rajaraman S., Gribok A., Ward W.K. (2007). Predictive monitoring for improved management of glucose levels. J. Diabetes Sci. Technol..

[3] Cobelli C., Dalla Man C., Sparacino G., Magni L., Kovatchev B.P. (2009). Diabetes, Models, Signals and Control. IEEE Rev. Biomed. Eng..

[4] Naumova V., Pereverzyev S.V., Sivananthan S. (2012). A meta-learning approach to the regularized learning—Case study: Blood glucose prediction. Neural Netw..

[5] Cappon G., Acciaroli G., Vettoretti M., Facchinetti A., Sparacino G. (2017). Wearable continuous glucose monitoring sensors: A revolution in diabetes treatment. Electronics.

[6] Rahmani A.M., Gia T.N., Negash B., Anzanpour A., Azimi I., Jiang M., Liljeberg P. (2018). Exploiting smart e-Health gateways at the edge of healthcare Internet-of-Things: A fog computing approach. Future Gener. Comput. Syst..

[7] Kafalı Ö., Bromuri S., Sindlar M., van der Weide T., Aguilar Pelaez E., Schaechtle U., Stathis K. (2013). Commodity 12: A smart e-health environment for diabetes management. J. Ambient Intell. Smart Environ..

[8] Rodríguez-Rodríguez I., Zamora-Izquierdo M.Á., Rodríguez J.V. (2018). Towards an ICT-based platform for type 1 diabetes mellitus management. Appl. Sci..

[9] Li K., Daniels J., Liu C., Herrero-Vinas P., Georgiou P. (2019). Convolutional recurrent neural networks for glucose prediction. IEEE J. Biomed. Health Inform..

[10] Marling C., Xia L., Bunescu R., Schwartz F. (2016). Machine learning experiments with noninvasive sensors for hypoglycemia detection. Proceedings of the IJCAI Workshop on Knowledge Discovery in Healthcare Data.

[11] Aiello E.M., Toffanin C., Messori M., Cobelli C., Magni L. (2019). Postprandial Glucose Regulation via KNN Meal Classification in Type 1 Diabetes. IEEE Control Syst. Lett..

[12] Zhu T., Li K., Herrero P., Chen J., Georgiou P. A Deep Learning Algorithm for Personalized Blood Glucose Prediction. Proceedings of the KHD@ IJCAI.

[13] Palumbo P., Ditlevsen S., Bertuzzi A., De Gaetano A. (2013). Mathematical modeling of the glucose–insulin system: A review. Math. Biosci..

[14] Pérez-Gandía C., Facchinetti A., Sparacino G., Cobelli C., Gómez E.J., Rigla M., Hernando M.E. (2010). Artificial neural network algorithm for online glucose prediction from continuous glucose monitoring. Diabetes Technol. Ther..

[15] Plis K., Bunescu R.C., Marling C., Shubrook J., Schwartz F. A machine learning approach to predicting blood glucose levels for diabetes management. Proceedings of the Workshops at the Twenty-Eighth AAAI Conference on Artificial Intelligence.

[16] Hamdi T., Ali J.B., Di Costanzo V., Fnaiech F., Moreau E., Ginoux J.M. (2018). Accurate prediction of continuous blood glucose based on support vector regression and differential evolution algorithm. Biocybern. Biomed. Eng..

[17] Sudharsan B., Peeples M., Shomali M. (2014). Hypoglycemia prediction using machine learning models for patients with type 2 diabetes. J. Diabetes Sci. Technol..

[18] Kane M.J., Price N., Scotch M., Rabinowitz P. (2014). Comparison of ARIMA and Random Forest time series models for prediction of avian influenza H5N1 outbreaks. BMC Bioinform..

[19] Sivananthan S., Naumova V., Man C.D., Facchinetti A., Renard E., Cobelli C., Pereverzyev S.V. (2011). Assessment of blood glucose predictors: The prediction-error grid analysis. Diabetes Technol. Ther..

[20] Naumova V., Nita L., Poulsen J.U., Pereverzyev S.V. (2016). Meta-learning based blood glucose predictor for diabetic smartphone app. Prediction Methods for Blood Glucose Concentration.

[21] Taie M., El-Faramawy I., Elmawazini M. (2015). Methods for Prediction, Simulation and Verification of Real-Time Software Architectural Design Based on Machine Learning Algorithms.

[22] Williams N., Zander S., Armitage G. (2006). A preliminary performance comparison of five machine learning algorithms for practical IP traffic flow classification. ACM SIGCOMM Comput. Commun. Rev..

[23] Douglas P.K., Harris S., Yuille A., Cohen M.S. (2011). Performance comparison of machine learning algorithms and number of independent components used in fMRI decoding of belief vs. disbelief. Neuroimage.

[24] Vink J.P., de Haan G. (2015). Comparison of machine learning techniques for target detection. Artif. Intell. Rev..

[25] Dubosson F., Ranvier J.E., Bromuri S., Calbimonte J.P., Ruiz J., Schumacher M. (2018). The open D1NAMO dataset: A multi-modal dataset for research on non-invasive type 1 diabetes management. Inform. Med. Unlocked.

[26] Box G.E., Jenkins G.M., Reinsel G.C., Ljung G.M. (2015). Time Series Analysis: Forecasting and Control.

[27] Breiman L. (1996). Bagging predictors. Mach. Learn..

[28] Vapnik V. (2013). The Nature of Statistical Learning Theory.

[29] Herrera D., Chen H., Lavoie E., Hendren L. (2018). Numerical computing on the web: Benchmarking for the future. Proceedings of the 14th ACM SIGPLAN International Symposium on Dynamic Languages.

[30] Toosi A.N., Son J., Buyya R. (2018). CLOUDS-Pi: A low-cost raspberry-pi based micro data center for software-defined cloud computing. IEEE Cloud Comput..

[31] Van Cauter E., Polonsky K.S., Scheen A.J. (1997). Roles of circadian rhythmicity and sleep in human glucose regulation 1. Endocr. Rev..

[32] Frandes M., Timar B., Timar R., Lungeanu D. (2017). Chaotic time series prediction for glucose dynamics in type 1 diabetes mellitus using regimeswitching models. Sci. Rep..

